# A case of ocular diffuse large B-cell lymphoma unveiled through Pars Plana Vitrectomy: surgical insight and multidisciplinary management

**DOI:** 10.1093/jscr/rjae399

**Published:** 2024-06-11

**Authors:** Pareena Sharma, Mustapha Tahiru, Han Li, Asha Bansari, Naveen Baskaran

**Affiliations:** Medical College of Georgia, Department of Medicine, Augusta, GA 30912, United States; Ohio State College of Medicine, Department of Medicine, Columbus, OH 43210, United States; University of Florida College of Medicine, Department of Medicine, Gainesville, FL 32610, United States; Department of Hospital Medicine, University of Florida Department of Hospital Medicine, Gainesville, FL 32610, United States; Department of Hospital Medicine, University of Florida Department of Hospital Medicine, Gainesville, FL 32610, United States

**Keywords:** lymphoma, vitrectomy, vitreoretinal, oncology, ophthalmology

## Abstract

We present a rare case of ocular diffuse large B-cell lymphoma (DLBCL) unveiled through Pars Plana Vitrectomy in a 74-year-old immunocompromised male, highlighting the surgical insight and multidisciplinary management required for similar cases. The patient’s progressive vision impairment led to a Pars Plana Vitrectomy, which confirmed DLBCL through cytogenetic analysis. Initial intraocular rituximab treatment showed promise; however, the lymphoma’s systemic progression necessitated a shift to more aggressive chemotherapy, underscored by pleural and pericardial effusions and central nervous system involvement. This case emphasizes the critical role of surgical techniques in diagnosing ocular lymphomas and the importance of a multidisciplinary approach in managing the disease’s ocular and systemic manifestations. The complexities introduced by the patient’s immunosuppression highlight the necessity for individualized treatment strategies. This case calls for further research into ocular lymphomagenesis and exploring therapies with enhanced efficacy and reduced toxicity, and emphasizes the importance of early diagnosis in ocular DLBCL cases.

## Introduction

Diffuse large B-cell lymphoma (DLBCL) accounts for approximately 25% of non-Hodgkin lymphomas cases [1]. Intraocular lymphoma is rare, comprising less than 1% of all non-Hodgkin lymphomas [2]. In the context of primary CNS lymphoma, a subset of patients—ranging from 15 to 25%—eventually develop vitreoretinal involvement, adding a layer of complexity to their clinical management [2]. In cases with primary vitreoretinal lymphoma (PVRL), a primary CNS lymphoma where the retina, subretinal pigment epithelium, and optic nerve can be involved, brain involvement increases to 56%- 90% [1, 2]. PVRL is generally classified as a DLBCL that is high grade. Primary DLBCL can also manifest as orbital lymphoma involving the orbit, choroid, ciliary body and iris [1]. The primary risk factors association for developing ophthalmic DLBCL is an older age of over 60 years old and immunocompromised status [3]. That same study concluded that PVRL was associated with lower survival rates, and the total resection of the malignancy was associated with this risk being decreased [1].

We present a case of DLBCL that manifested post-vitrectomy in a 74-year-old male. This case underscores the rarity of ocular DLBCL presentations and emphasizes the intricacies involved in its diagnosis and treatment, particularly in the realm of ophthalmology. We delve into the clinical manifestations of ocular DLBCL, its sampling techniques during surgery and the challenges encountered in its management within the ophthalmology clinic.

## Case report

**Figure 1:**
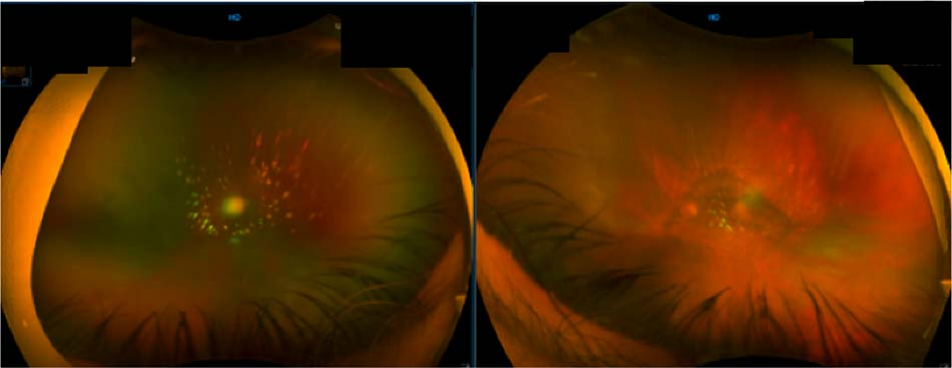
Fundal image of right and left eye. Right eye: large PVD with opacification and some vitreous cells. Left eye: nuclear sclerosis of intraocular lens and large PVD with opacification.

This case involves a 74-year-old male with a medical history of atrial fibrillation and a liver transplant for alcohol-associated cirrhosis, who presented with progressive vision impairment characterized by bilateral floaters, flashes and photophobia severely affecting his professional abilities as a neuroradiologist. This impairment was attributed to bilateral posterior vitreous detachment (PVD) with opacification (Fig. 1). The complexity of his case was heightened by his post-transplant immunosuppressive regimen, consisting of mycophenolate and tacrolimus.

The initial surgical intervention involved a pars plana vitrectomy (PPV). This procedure involved the removal of the vitreous gel from the posterior segment of the eye to alleviate the patient’s symptoms and secure a vitreous sample for diagnostic purposes. The subsequent flow cytometry analysis of the vitreous sample revealed CD20-positive B cells with kappa light chain restriction, a finding indicative of aggressive non-Hodgkin’s B-cell lymphoma. This diagnosis was further supported by comprehensive cytogenetics and FISH analyses, which confirmed the absence of common lymphoma-associated genetic alterations, thus affirming the diagnosis of DLBCL. MRI of the orbit and brain did not show pathological change in the intra-orbital regions and PET-CT showed no findings to suggest systemic lymphoma.

**Figure 2:**
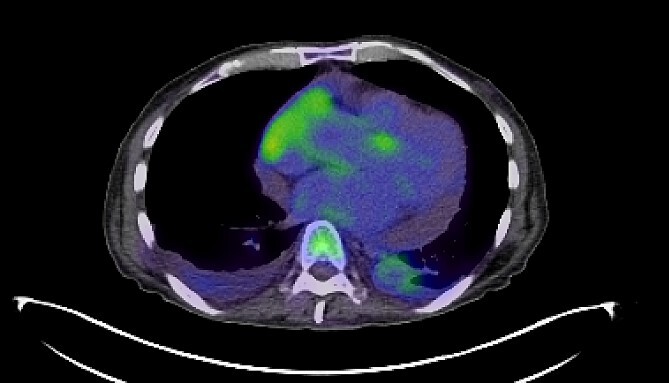
PET-CT imaging indicating pericardial and pleural involvement of lymphoma.

In response to the initial intraocular lymphoma diagnosis, the patient received intraocular rituximab treatment with a promising initial response. However, the subsequent emergence of fevers, chills, night sweats and muscle weakness with evidence of pleural and pericardial effusions necessitated additional interventions, including pericardiocentesis and thoracentesis, revealing the systemic progression of the lymphoma. Pericardiocentesis and thoracentesis both showed aggressive lymphoma that was CD20-, CD38- and CD45-positive and CD5-, CD10- and CD11C-negative. KI67 was increased in 50% of cells. PET-CT showed FDG-AVID right-sided pleural effusion and moderate-large pericardial effusion with foci of mild FDG uptake anteriorly (Fig. 2). He was treated with three cycles of R-EPOCH that was stopped after the patient experienced severe fatigue and weakness. PET again showed no evidence of disease.

**Figure 3:**
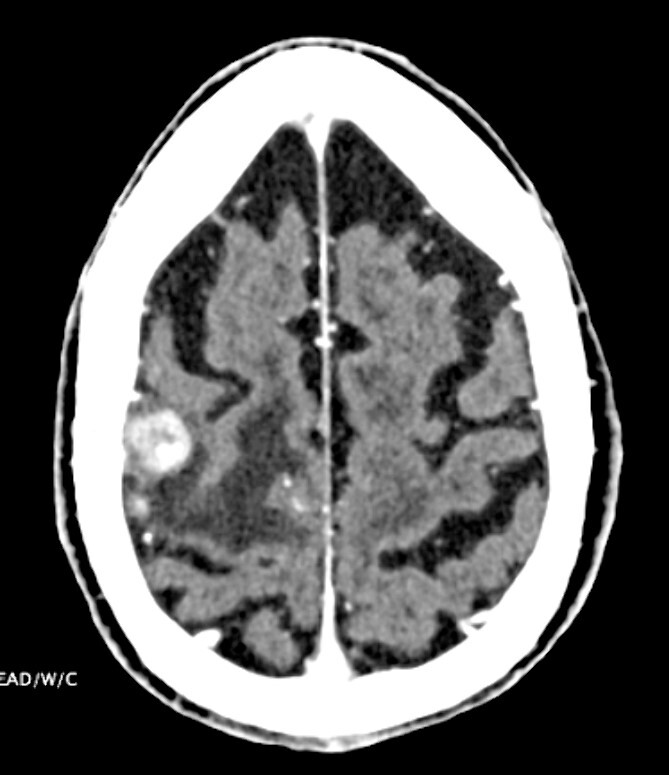
CT head indicating 1.3-cm right frontal lobe motor cortex heterogeneously enhancing lesion.

Six months later, the patient had an episode of left-sided focal motor seizure. CT head showed a 1.3-cm heterogeneously enhancing mass lesion with the right frontal lobe motor cortex (Fig. 3). Follow-up MRI-brain revealed several well-defined enhancing nodular lesions within the right frontal lobe predominantly in the region of the pericentral gyrus, the largest of which measured 1.8 cm. The whole body PET-CT showed focal intense activity in the left frontal lobe of the brain corresponding to the area seen on CT and MRI. The patient was started on levetiracetam, dexamethasone, high-dose methotrexate and rituximab. He was treated with high-dose methotrexate and rituximab for presumed recurrence of non-Hodgkin’s lymphoma with plans for three cycles followed by repeat MRI.

## Discussion

The presented case underscores the critical importance of a multidisciplinary approach in diagnosing and treating ocular manifestations of lymphoma, particularly in patients with ongoing immunosuppression. Ocular lymphomas, including PVRL, pose unique diagnostic and therapeutic challenges due to their rarity and varied clinical presentations.

The diagnostic suspicion was raised with the initial ophthalmological evaluation due to progressive vision impairment (Fig. 1). The subsequent PPV allowed for the collection of vitreous samples, which confirmed the diagnosis of DLBCL through flow cytometry and comprehensive cytogenetic analyses. This highlights the pivotal role of surgical techniques and sampling methods in achieving accurate diagnoses in ocular lymphomas.

Treatment strategies for PVRL range from systemic high-dose methotrexate, chemotherapy adjunct with stem-cell transplant, and single-agent treatments like ibrutinib, lenalidomide and temozolomide [4]. In this case, intraocular rituximab was initially employed to target CD20-positive B cells within the eye. However, the systemic progression of DLBCL, as evidenced by pleural and pericardial effusions and later CNS involvement, necessitated high-dose methotrexate and rituximab to address CNS disease burden, underscoring the aggressive nature of the disease and the challenges in achieving disease control (Figs 2 and 3).

Despite advancements in diagnostic techniques, PVRL diagnosis can be delayed because it may present similarly to uveitis with an initial response to steroid therapy [4]. However, it is important to diagnose PVRL early in its course due to risk of permanent vision loss, CNS relapse and death [4]. Moreover, cytology sample can be challenging to obtain due to the fragility of the lymphoma cells in the vitreous [4]. Furthermore, the complexities introduced by concomitant medical conditions, such as immunosuppression following organ transplantation, necessitate careful consideration in treatment planning and monitoring.

Research should focus on underlying mechanisms of ocular lymphomagenesis, exploring therapies with enhanced efficacy and reduced toxicity and optimizing treatment strategies for patients with systemic disease progression. Additionally, larger prospective studies are warranted to better delineate the epidemiology, prognostic factors and long-term outcomes of ocular DLBCL. This case highlights the intricacies involved in diagnosing and managing ocular DLBCL and emphasizes the necessity for a multidisciplinary approach to treating ocular manifestations of lymphoma.

## Conflict of interest statement

None declared.

## Funding

None declared.
